# Epigenetic Dysregulation in Neurodegenerative Disease: Implications for Neuropathology and Therapy

**DOI:** 10.7759/cureus.90188

**Published:** 2025-08-15

**Authors:** Hussein Qasim, Karis Khattab, Mohammad Abu Shugaer, Giustino Varrassi

**Affiliations:** 1 Pathology and Laboratory Medicine, Jordan University of Science and Technology, Irbid, JOR; 2 Pathology and Laboratory Medicine, Faculty of Medicine, Yarmouk University, Irbid, JOR; 3 Pain Medicine, Fondazione Paolo Procacci, Rome, ITA

**Keywords:** alzheimer’s disease, biomarkers, epigenetics, huntington’s disease, neurodegeneration, parkinson’s disease

## Abstract

Neurodegenerative diseases (NDDs) such as Alzheimer’s disease (AD), Parkinson’s disease (PD), and Huntington’s disease (HD) are characterized by progressive neuronal dysfunction, yet their underlying mechanisms remain incompletely understood. Emerging evidence implicates epigenetic dysregulation as a central contributor to the pathogenesis of these disorders. A thematic literature review was conducted across major databases using targeted search terms related to epigenetics and neurodegeneration. Studies were selected based on relevance, methodological quality, and contribution to mechanistic understanding, in accordance with Scale for the Assessment of Narrative Review Articles (SANRA) guidelines. Across AD, PD, and HD, distinct yet overlapping patterns of epigenetic alterations were identified. In AD, dysregulated DNA methylation and histone acetylation affect genes linked to amyloid and tau pathology. In PD, hypomethylation of SNCA and altered histone acetylation contribute to α-synuclein overexpression and neuronal loss. In HD, mutant huntingtin protein disrupts chromatin remodeling by sequestering histone acetyltransferases and altering microRNA expression. These changes disrupt neuronal identity, synaptic function, and inflammatory responses, often forming feedback loops that exacerbate disease progression. Epigenetic mechanisms play a pivotal role in neurodegeneration by mediating gene-environment interactions and perpetuating neuropathological changes. Their reversible nature presents opportunities for therapeutic intervention, though challenges related to specificity, delivery, and timing remain. Continued research into epigenetic biomarkers and precision-targeted epigenetic therapies holds promise for advancing early diagnosis and disease modification in NDDs.

## Introduction and background

Neurodegenerative diseases (NDDs) such as Alzheimer’s disease (AD), Parkinson’s disease (PD), and Huntington’s disease (HD) are characterized by progressive neuronal loss and accumulation of misfolded proteins, leading to cognitive and motor impairment [1]. Despite intense research, effective disease-modifying treatments remain elusive, partly due to the multifactorial nature of these disorders [2]. Both genetic predispositions and environmental exposures contribute to NDD pathogenesis, and recent work highlights epigenetics as a critical interface between these factors [3]. Epigenetics refers to heritable yet reversible modifications of gene function that do not involve changes in DNA sequence, including DNA methylation, histone modifications, and non-coding RNA activity [4]. These mechanisms are central to normal brain development and plasticity, but their dysregulation can bridge life-long environmental influences (toxins, diet, and stress) with gene expression changes that drive neurodegeneration [5]. Evidence accumulated over the past two decades implicates epigenetic dysregulation in the pathology of AD, PD, HD, and other NDDs [6]. Aberrant DNA methylation patterns, altered post-translational modifications of histone proteins, dysregulated microRNA, and long non-coding RNA profiles have been observed in affected brains [7]. These epigenetic changes can lead to inappropriate silencing or activation of genes involved in neuronal survival, synaptic function, inflammation, and protein homeostasis [7].

In many cases, toxic protein aggregates characteristic of NDDs can themselves perturb epigenetic enzymes; for example, α-synuclein and mutant huntingtin protein have been shown to sequester or inhibit key epigenetic regulators [8]. Such findings suggest a vicious cycle whereby neuropathological lesions induce epigenomic aberrations that further compromise neuronal health [9]. Given that epigenetic marks are potentially reversible, understanding these processes opens new avenues for therapeutic intervention [10]. In this review, we synthesize current knowledge of epigenetic mechanisms in the context of AD, PD, and HD, highlighting molecular and pathological insights from human studies and animal models. We also discuss how epigenetic changes correlate with neuropathological features and consider emerging epigenome-targeting therapies, along with the challenges and future directions for translating these insights into clinical advances.

## Review

Methodology

This narrative review employs a comprehensive descriptive thematic analysis to examine the role of epigenetic dysregulation in the pathogenesis and progression of major NDDs, specifically AD, PD, and HD. The review adheres to the Scale for the Assessment of Narrative Review Articles (SANRA) guidelines to ensure scientific rigor, transparency, and academic credibility. Included studies were peer-reviewed articles focusing on epigenetic mechanisms, such as DNA methylation, histone modification, and non-coding RNAs, in the context of AD, PD, or HD. Eligible literature comprised original research studies, systematic reviews, meta-analyses, and narrative reviews. Preclinical studies, including animal models and in vitro experiments, were considered if they provided mechanistic insight relevant to human disease. Editorials, commentaries, and conference abstracts were excluded unless they presented novel hypotheses or significant conceptual frameworks. Studies were excluded if they lacked clear relevance to epigenetics in neurodegeneration or did not offer full-text access. A comprehensive literature search was conducted using PubMed, Scopus, Web of Science, Embase, and Google Scholar. The search covered literature published from January 2000 to March 2025. The search was performed using a combination of Medical Subject Headings (MeSH) and free-text terms such as “epigenetics AND Alzheimer’s disease,” “DNA methylation AND Parkinson’s disease,” “histone modifications AND Huntington’s disease,” and “non-coding RNAs AND neurodegeneration.” Boolean operators “AND” and “OR” were employed to refine results. The search was extended to include synonyms and related terms, such as “chromatin remodeling,” “microRNAs,” “lncRNAs,” “neuroepigenetics,” and “neurodegenerative disorders." References were managed using EndNote. Two independent reviewers screened titles and abstracts based on the eligibility criteria. Full-text articles were subsequently assessed for inclusion. Discrepancies were resolved through discussion and consensus, with arbitration by a third reviewer when necessary. The inter-reviewer agreement exceeded 85% in the pilot screening phase, indicating consistency in the selection methodology. Reference lists of included articles were also screened for additional relevant studies using backward citation tracking.

Overview of epigenetic mechanisms

Epigenetic regulation in the CNS governs gene expression without altering DNA sequence, primarily through DNA methylation, histone modifications, and non-coding RNAs, all of which play crucial roles in maintaining neuronal identity, function, and plasticity [11]. DNA methylation involves the addition of methyl groups to CpG sites, typically silencing genes by blocking transcription factor access or recruiting repressive complexes [12]. In neurons, dynamic methylation and its oxidized form, 5-hydroxymethylcytosine (5-hmC), are essential for synaptic function and memory [13]. Disruption of these patterns can lead to neuronal dysfunction and degeneration [13]. Histone modifications affect chromatin structure and gene accessibility [14]. Acetylation, mediated by histone acetyltransferases (HATs), typically activates transcription by loosening chromatin, while deacetylation by histone deacetylases (HDACs) represses it [15]. Methylation can signal either activation (e.g., H3K4me3) or repression (e.g., H3K9me3) [16]. These modifications coordinate with DNA methylation to regulate learning, memory, and neuron-specific gene expression [16]. Aberrant histone patterns, such as reduced acetylation of neuroprotective genes, are frequently observed in neurodegenerative conditions [17]. Non-coding RNAs, including microRNAs (miRNAs) and long non-coding RNAs (lncRNAs), modulate gene expression post-transcriptionally or via chromatin remodeling [18]. Brain-specific miRNAs like miR-132 regulate synaptic plasticity and memory [19]. In neurodegeneration, dysregulated miRNAs and lncRNAs can disrupt neuroprotective and anti-inflammatory pathways [20]. These RNAs are also being investigated as non-invasive biomarkers, as their expression in blood and cerebrospinal fluid (CSF) may reflect CNS pathology [21]. Together, these mechanisms create a dynamic and responsive epigenetic landscape in the brain, one that becomes maladaptive in NDDs.

Epigenetics in Alzheimer’s disease

AD, characterized by amyloid-β (Aβ) plaques and neurofibrillary tangles (NFTs), exhibits widespread epigenetic dysregulation that contributes to its pathogenesis [22]. Epigenetic changes in AD affect genes involved in amyloid processing, tau pathology, inflammation, and synaptic function [23].

Alterations in DNA methylation are a key feature of AD. Studies of postmortem AD brains reveal inconsistent global DNA methylation patterns, some showing decreased 5-methylcytosine (5-mC) levels and others increased, likely due to regional and cell-type variability [24]. More refined analyses demonstrate that NFT-bearing hippocampal neurons have reduced DNA methylation, linking methylation deficits to tau pathology [25]. In contrast, levels of 5-hmC are elevated in the AD brain and correlate with disease severity [26]. Locus-specific methylation changes are also consistently observed in AD [27]. For example, hypomethylation of the MAPT gene promoter is associated with increased tau expression and aggregation, while hypomethylation of BACE1, a key gene in Aβ production, may exacerbate amyloid burden [28]. Conversely, the ANK1 gene, involved in immune regulation, is repeatedly found to be hypermethylated in AD brains, although its functional role remains unclear [29]. Large epigenome-wide association studies (EWAS) have identified numerous differentially methylated CpG sites in the AD cortex, many of which correlate with hallmark pathological features such as plaques and tangles [30]. These findings suggest that both hypo- and hypermethylation at specific genomic loci contribute to the disrupted gene expression programs that underlie AD progression [31].

Chromatin remodeling plays a critical role in gene expression changes associated with cognitive decline in AD [32]. A hallmark of AD brains is the downregulation of synaptic plasticity genes and upregulation of inflammatory or stress-response genes, regulated in part by histone modifications [33]. Notably, there is widespread loss of histone acetylation, especially H3K27ac, at enhancers and promoters of memory-related genes (e.g., BDNF (brain-derived neurotrophic factor) and CREB targets) in brain regions such as the entorhinal cortex [34]. This loss correlates with decreased gene expression and impaired cognition [35]. One contributing factor is the elevation of HDAC2, which represses transcription by removing acetylation marks from neuronal genes [15]. In AD mouse models, HDAC2 overexpression leads to synaptic dysfunction, while treatment with HDAC inhibitors restores histone acetylation, reactivates gene expression, and improves memory [36]. Other histone marks are also dysregulated: repressive modifications like H3K9me3 and H3K27me3 are elevated at neuronal gene loci, contributing to transcriptional silencing [37]. Activation marks such as H3K27ac may appear at promoters of pro-inflammatory genes; for example, hyperacetylation of the IL-1β promoter in microglia promotes chronic neuroinflammation in AD [38]. Together, these findings suggest a global reconfiguration of chromatin architecture in AD, driven by altered activity of chromatin-modifying enzymes including HDACs, HATs, and methyltransferases.

Non-coding RNAs also play a major role in the epigenetic regulation of AD pathology [39]. Both miRNAs and lncRNAs are dysregulated in AD brains and biofluids [18]. Several miRNAs associated with synaptic function and neuroinflammation are altered. For example, miR-132, essential for synaptic integrity, is consistently downregulated in AD, which contributes to tau pathology and cognitive impairment by disinhibiting pro-inflammatory and tau-related gene expression [40,41]. In contrast, miR-146a is upregulated, particularly in glial cells, where it may initially serve an anti-inflammatory role but becomes maladaptive with chronic elevation [42]. Additional miRNAs such as miR-29 and miR-107, which normally suppress BACE1 (a key Aβ-producing gene), are also reduced, potentially increasing amyloidogenic processing [43]. Others, including miR-125b and miR-146, contribute to inflammatory signaling in glia [43]. In terms of lncRNAs, several transcripts show dysregulation in AD. BACE1-AS, which stabilizes BACE1 mRNA, may promote Aβ accumulation, while NEAT1, associated with stress granule formation and inflammation, is also upregulated [44]. Notably, some miRNAs show promise as biomarkers; altered levels of miR-125b and miR-142-3p in blood and CSF have been reported to differentiate AD patients from healthy controls [45]. These findings highlight the potential of non-coding RNAs as both mechanistic contributors and diagnostic tools in AD.

Epigenetics in Parkinson’s disease

PD is characterized by dopaminergic neuron loss in the substantia nigra and α-synuclein aggregation in Lewy bodies [46]. While some cases are linked to mutations (e.g., SNCA, LRRK2, and PINK1), most are sporadic, involving environmental triggers [47]. Epigenetic mechanisms, DNA methylation, histone modifications, and non-coding RNAs, play key roles in translating environmental and genetic risk into lasting molecular changes in the brain [11].

DNA Methylation

Hypomethylation of SNCA intron 1 is a consistent finding in PD neurons, leading to increased α-synuclein expression [48]. Other PD-related genes, such as PINK1, PRKN, DJ-1, and PGC-1α, also show aberrant methylation patterns that correspond with mitochondrial dysfunction and oxidative stress [49]. Notably, α-synuclein can mislocalize the DNA methyltransferase DNMT1 from the nucleus to the cytoplasm, leading to global hypomethylation in PD neurons [50], highlighting a feedback loop between protein aggregation and epigenetic disruption.

Histone Modifications

PD neurons often show decreased histone acetylation, especially at genes important for dopamine synthesis and mitochondrial defense [51]. HDAC upregulation after neurotoxin exposure worsens neuronal damage, while HDAC inhibitors like sodium butyrate offer neuroprotection in models [52]. Additionally, repressive histone methylation (e.g., H3K9me) is elevated in PD neurons, contributing to transcriptional silencing [53]. Enzymes like SIRT1 and SIRT2 modulate these effects, with opposing roles in mitochondrial regulation and neuroprotection [54]. Several miRNAs critical for dopaminergic neuron maintenance are altered in PD. miR-133b and miR-34b/c are reduced in PD brain tissue, leading to impaired neuronal survival and increased α-synuclein [55]. Others, like miR-126, are elevated and may enhance vulnerability to apoptosis [56]. Peripheral miRNA changes mirror brain alterations, suggesting potential for biomarker development [57]. lncRNAs such as NEAT1, MALAT1, and HOTAIR are also dysregulated, potentially affecting chromatin states and stress responses [58].

Epigenetics in Huntington’s disease

HD is a monogenic neurodegenerative disorder caused by an expanded CAG repeat in the HTT gene, resulting in mutant huntingtin (mHTT) protein [59]. Despite its genetic origin, HD involves widespread epigenetic dysregulation, making it a model for understanding epigenetic contributions to neurodegeneration [60].

Histone Acetylation and Chromatin Dysregulation

mHTT binds and sequesters CREB-binding protein (CBP), a HAT essential for activating genes involved in neuroplasticity and survival [61]. This leads to reduced histone acetylation and transcriptional repression of neuron-specific genes such as BDNF, leading to impaired synaptic and metabolic functions in HD neurons [62]. Preclinical models have shown that HDAC inhibitors (e.g., sodium butyrate and SAHA) can partially restore acetylation, improve motor symptoms, and delay progression, although toxicity limits their translational use [63].

Histone Methylation and Chromatin Silencing

mHTT enhances gene repression by interacting with polycomb repressive complex 2 (PRC2), increasing H3K27me3 marks [64]. Other chromatin-modifying enzymes like SETDB1 (H3K9 methyltransferase) and KDM5C (H3K4 demethylase) are also dysregulated in HD, further promoting transcriptional silencing [65]. Additionally, mHTT impairs the regulation of REST, a transcriptional repressor, allowing it to enter the nucleus and silence many neuronal genes and miRNAs, compounding the loss of neuronal identity [66]. Though less prominent than histone changes, HD brains show gene-specific DNA methylation alterations, particularly at genes involved in synaptic function and development [67]. Aberrant one-carbon metabolism in HD may affect methyl donor availability (SAM), influencing methylation patterns, the principal methyl donor for DNA and histone methylation reactions, thereby influencing epigenetic regulation [68]. Some studies suggest that epigenetic interventions may induce heritable DNA methylation changes across generations in HD models [69]. Moreover, numerous miRNAs are dysregulated in HD, including REST-repressed miR-124, miR-132, and miR-212, all downregulated and linked to impaired synaptic function [70]. Others, like miR-146a, are upregulated in response to inflammation [71]. Additionally, HD affects miRNA processing components (e.g., Dicer and argonaute), causing global miRNA imbalance [72]. Dysregulation of lncRNAs also suggests lncRNAs may modulate neuronal gene expression in HD [73].

Feedback loops across diseases

**Figure 1:**
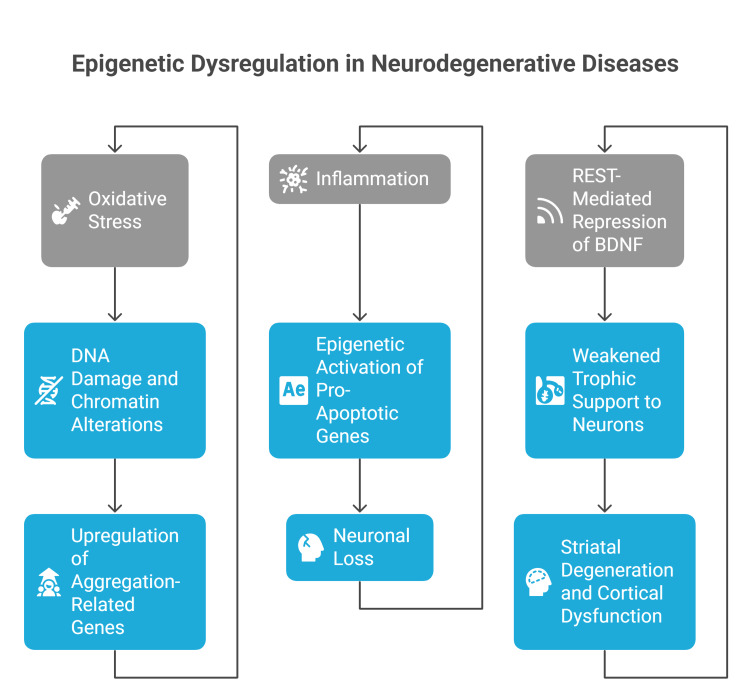
Feedback loops linking epigenetic dysregulation with neuropathology in NDDs. NDDs, neurodegenerative diseases; BDNF, brain-derived neurotrophic factor The figure was created by the authors.

NDDs share common feedback loops between epigenetic dysregulation and pathological progression [74]. As illustrated in Figure 1, these feedback mechanisms contribute to the advancement of AD, PD, and HD through distinct yet overlapping pathways. In AD, oxidative stress resulting from Aβ and tau accumulation leads to DNA damage and chromatin alterations, which subsequently upregulate genes that promote further protein aggregation [75]. In PD, inflammation triggered by Lewy body formation activates pro-apoptotic genes via epigenetic modifications, thereby accelerating neuronal loss [76]. In HD, repression of BDNF by the transcriptional repressor REST diminishes trophic support to neurons, hastening striatal degeneration and potentially contributing to cortical dysfunction through feedback effects [77].

Therapeutic implications

Epigenetic alterations in NDDs are increasingly viewed as viable therapeutic targets due to their reversible nature [78]. Various epigenetic drugs, many adapted from oncology, have shown promise in preclinical models of AD, PD, and HD disease [78]. HDAC inhibitors are the most extensively studied [79]. In AD models, drugs like sodium butyrate and SAHA restore histone acetylation, improve cognition, and reduce amyloid and tau pathology [80]. Similar neuroprotective effects have been seen in PD and HDs [80]. Early-phase clinical trials have shown modest cognitive improvements, though long-term efficacy and safety remain uncertain [81]. Challenges include off-target effects, systemic toxicity, and limited brain penetration [82]. To address these, research is focusing on isoform-selective and brain-targeted HDAC inhibitors [83]. DNA methylation modulators like DNMT inhibitors (e.g., 5-azacytidine) can reactivate silenced genes but are not yet viable for brain disorders due to toxicity and lack of CNS specificity [84]. Nutritional approaches, such as B-vitamin and SAM supplementation, offer indirect modulation of methylation and have shown neuroprotective effects in some studies [85]. Non-coding RNA-based therapies are another emerging area [86]. Strategies include miRNA mimics (e.g., miR-132 in AD) and antagomirs (e.g., targeting miR-146a) [87]. In PD and HD, approaches to reduce alpha-synuclein or mutant HTT via RNA targeting or CRISPR-based epigenome editing are in development [88]. These offer high specificity and potential for disease modification. Emerging epigenetic technologies like CRISPR-dCas9 fusion systems allow locus-specific activation or repression of genes without altering the DNA sequence [89]. Though conceptually powerful, these face delivery and safety challenges before clinical application [90]. Also, epigenetic drugs may enhance the efficacy of existing treatments, e.g., HDAC inhibitors might complement immunotherapies in AD or dopamine therapies in PD [91]. HDAC inhibitors may also promote regeneration when combined with stem cell or neurotrophic factor therapies [92]. Moreover, lifestyle factors like exercise and diet, which exert epigenetic effects, may offer low-risk complementary strategies [93]. For example, exercise increases BDNF expression via histone acetylation [94]. Finally, the overcorrection or off-target epigenetic effects could be harmful, potentially activating oncogenes or affecting non-neuronal tissues [95]. Early intervention, before extensive neuronal loss, is likely key [96]. Biomarker-guided patient selection may help optimize timing and targeting [97].

Research gaps and future directions

While progress has been made in neuroepigenetics, several important questions remain [98]. A key issue is whether epigenetic changes are a cause of neurodegeneration or a consequence [99]. Experimental models with controlled epigenetic alterations are needed to clarify this [100]. Another major priority is cell-type-specific analysis: bulk tissue studies can obscure important differences, so techniques like single-cell sequencing and sorted nuclei profiling should be used to map epigenetic changes in vulnerable neuronal populations [101]. Integrating epigenetic data with transcriptomics, proteomics, and genetics will help uncover causal mechanisms and regulatory networks [102]. Similarly, studying how environmental factors such as toxins, diet, or exercise influence the epigenome could lead to preventative strategies [103]. Epigenetic biomarkers, like DNA methylation signatures or miRNA panels, also hold potential for early diagnosis and tracking disease progression, but they need further validation and refinement, especially for brain specificity and clinical applicability [104]. Finally, deeper mechanistic studies are required to connect specific epigenetic changes with neuronal dysfunction [105]. Exploring epigenetic alterations across multiple NDDs may reveal shared pathways and targets for intervention [99]. Bridging these gaps will require collaboration across molecular biology, bioinformatics, and clinical research to realize the full therapeutic potential of epigenetics in neurodegeneration [106]. The use of machine learning and artificial intelligence might definitely improve the clinical process [107,108].

## Conclusions

Epigenetic dysregulation, through changes in DNA methylation, histone modifications, and non-coding RNAs, has emerged as a central mechanism linking genetic risk, environmental exposures, and neuropathology in major NDDs such as AD, PD, and HD. These changes can silence protective genes and activate degenerative pathways, contributing directly to disease progression. Importantly, epigenetic marks are reversible, making them attractive therapeutic targets and potential early biomarkers for diagnosis and patient stratification. Although still in early stages, epigenetic therapies show promise in modifying disease trajectories by simultaneously addressing multiple pathogenic pathways. Continued interdisciplinary research is essential to translate these findings into safe and effective treatments, positioning epigenetics as a promising frontier in combating neurodegeneration.
